# A comparative analysis of conventional and speckle-tracking strain echocardiographic findings in diabetic and non-diabetic kidney disease patients with normal ejection fraction

**DOI:** 10.1007/s10554-022-02687-9

**Published:** 2022-07-22

**Authors:** Ganesh Paramasivam, Indu Ramachandra Rao, Jyothi Samanth, Krishnananda Nayak, Rakshitha Nayak, Simran Agnes Martis, Rinkle Jerome, Shankar Prasad Nagaraju, Ravindra Attur Prabhu, Tom Devasia

**Affiliations:** 1 Department of Cardiology, Kasturba Medical College, Manipal, Manipal Academy of Higher Education, Manipal, 576104 Karnataka India; 2 Department of Nephrology, Kasturba Medical College, Manipal, Manipal Academy of Higher Education, Manipal, 576104 Karnataka India; 3 Department of Cardiovascular Technology, Manipal College of Health Professions, Manipal Academy of Higher Education, Manipal, 576104 Karnataka India

**Keywords:** Strain echocardiography, Strain imaging, Deformation imaging, Speckle-tracking, Diastolic dysfunction, Diabetic kidney disease, Diabetic nephropathy, Chronic kidney disease, Atherosclerotic cardiovascular disease, Major adverse cardiovascular events, Cardiovascular outcomes

## Abstract

This study aimed to compare the differences in echocardiographic and strain parameters in patients with diabetic kidney disease (DKD) and non-diabetic kidney disease (NDKD) in a cohort with pre-dialysis chronic kidney disease (CKD) and normal ejection fraction (EF). In this single-center prospective study, patients with CKD stages 3–5 and EF > 55% were included. We compared cardiac structure and function using conventional and speckle-tracking strain echocardiography among DKD and NDKD groups. Cardiovascular outcomes were assessed at the end of the study. Of the included 117 patients, 56 (47.9%) had DKD, and 61 (52.1%) had NDKD. Patients with DKD had higher ratios of early mitral inflow velocity and mitral annular early diastolic velocity (E/e’) (11.9 ± 4.4 vs. 9.8 ± 3.5; *p* = 0.004), lower septal e’ velocity (7.1 ± 2.5 vs. 8.2 ± 2.8; *p* = 0.031), lower lateral e’ velocity (9.2 ± 2.9 vs. 10.4 ± 3.8; *p* = 0.045) and longer deceleration times (209.2 ± 41.5 vs. 189.1 ± 48.0; *p* = 0.017), compared to those with NDKD. Left ventricular mass index (LVMI), global longitudinal strain (GLS), early diastolic strain rate (SR_E),_ and E/SR_E_ were similar. At a median follow-up of 239 days, 3-P MACE (11.5% vs. 4.9%; *p* = 0.047) and 4-P MACE (28.6% vs. 11.5%; *p* = 0.020) were observed to be higher in the DKD group. Diastolic dysfunction was more common in patients with DKD, compared to those with NDKD, although both groups had similar LVMI and GLS. Those with DKD also had poorer cardiovascular outcomes. This highlights the importance of the assessment of diastolic function in CKD, particularly in those with diabetic CKD.

## Introduction

Patients with chronic kidney disease (CKD) are at an increased risk of cardiovascular disease (CVD) compared to the general population. CVD is the most common cause of mortality in patients with CKD, accounting for 40–50% of deaths in those with advanced kidney disease [1, 2]. In addition to cardiovascular mortality related to atherosclerotic cardiovascular disease (ASCVD), mortality attributable to sudden cardiac death and heart failure is also significantly higher in CKD, compared to the general population [3].

Echocardiography is a simple, non-invasive, and affordable means of cardiovascular risk stratification in CKD. Studies have found that echocardiographic findings such as left ventricular hypertrophy (LVH), reduced ejection fraction (EF), and diastolic dysfunction predict future risk of adverse cardiovascular events and all-cause mortality [4–6]. There is also a growing body of evidence that deformation analysis by speckle-tracking echocardiography (also called strain imaging) can identify cardiac abnormalities earlier than conventional echocardiographic techniques. This modality could potentially transform our understanding of cardiomyopathy in CKD, help in prognostication, and improve therapeutic decision-making [7].

Of those with CKD, patients with diabetic kidney disease (DKD) form a distinct sub-group. These patients account for approximately 40% of the CKD population and are at a higher risk of cardiovascular events, compared to their counterparts with non-diabetic CKD [8]. Little is known, however, about the differences in echocardiographic and strain parameters between these two groups. We sought to compare both conventional echocardiographic and strain parameters in patients with DKD and those with non-diabetic kidney disease (NDKD).

## Methods

### Study design and setting

This was a prospective study conducted in the departments of Nephrology and Cardiology during the period March 2019 to Dec 2021 at Kasturba Medical College, Manipal, a tertiary care hospital in South India. The study was approved by the institutional ethics committee and was conducted by the declaration of Helsinki. The study was registered in the Clinical Trials Registry of India (CTRI/2019/03/018119).

### Study participants

Patients aged ≥ 40 years with CKD stages 3–5 and normal left ventricular ejection fraction (EF ≥ 55%) in sinus rhythm were included. CKD was diagnosed and staged as per the Kidney Disease: Improving Global Outcomes (KDIGO) guidelines [9]. Those with known coronary artery disease, significant valvular heart disease, congenital heart disease, pericardial disease causing significant pericardial effusion or chronic constrictive pericarditis, irregular rhythms like atrial fibrillation or flutter, the poor window for echocardiography, patients on hemodialysis or peritoneal dialysis, pregnant patients, seriously ill patients, and patients unable to lie down comfortably for the duration of the study were excluded.

### Study procedure

Eligible patients who were willing to participate were enrolled after obtaining written informed consent. A brief clinical history was taken, and baseline data were noted. Physical examination findings and baseline lab parameters were also recorded.

Based on the etiology of CKD, patients were grouped as either having DKD or non-diabetic kidney disease (NDKD) as follows:

DKD group: CKD patients with diabetes and (a) biopsy evidence of DKD or (b) presence of diabetic retinopathy and no clinical or serological evidence suggesting alternate causes of CKD.

NDKD group: (a) CKD patients without diabetes or (b) those with diabetes but biopsy evidence of other causes of CKD (in the absence of histopathological evidence of diabetic changes).

#### Echocardiographic assessment

Detailed echocardiographic evaluation was done using standard views including parasternal long axis and short axis view at three levels of the left ventricle and apical 4-, 3- and 2-chamber views. All echocardiographic studies were performed on the GE VIVID S60 2D echocardiographic system (GE healthcare, US) with 1.3–4.5 MHz 3Sc-RS adult probe. Echocardiographic images were stored for offline analysis. Left ventricular (LV) internal diameter during end diastole (LVEDD), LV internal diameter during end-systole (LVESD), wall thickness of posterior wall of LV (PWT), and interventricular septum (IVST) were recorded. Left ventricle ejection fraction measurements were taken by using M-Mode and Modified Simpson’s method. LV end diastolic volume (LVEDV) and LV end systolic volume (LVESV) were measured by tracing the border between myocardium and LV cavity as recommended in the 2015 American Society of Echocardiography/European Association of Cardiovascular Imaging (ASE/EACVI) guidelines [10]. LV mass (LVM) and LV mass index (LVMI) were calculated using Devereaux formula [11]. LV hypertrophy (LVH) was deemed to be present when LVMI was more than 95 g/m^2^ in women and 115 g/m^2^ in men.

#### Pulse-wave doppler and tissue doppler echocardiography

**Fig. 1:**
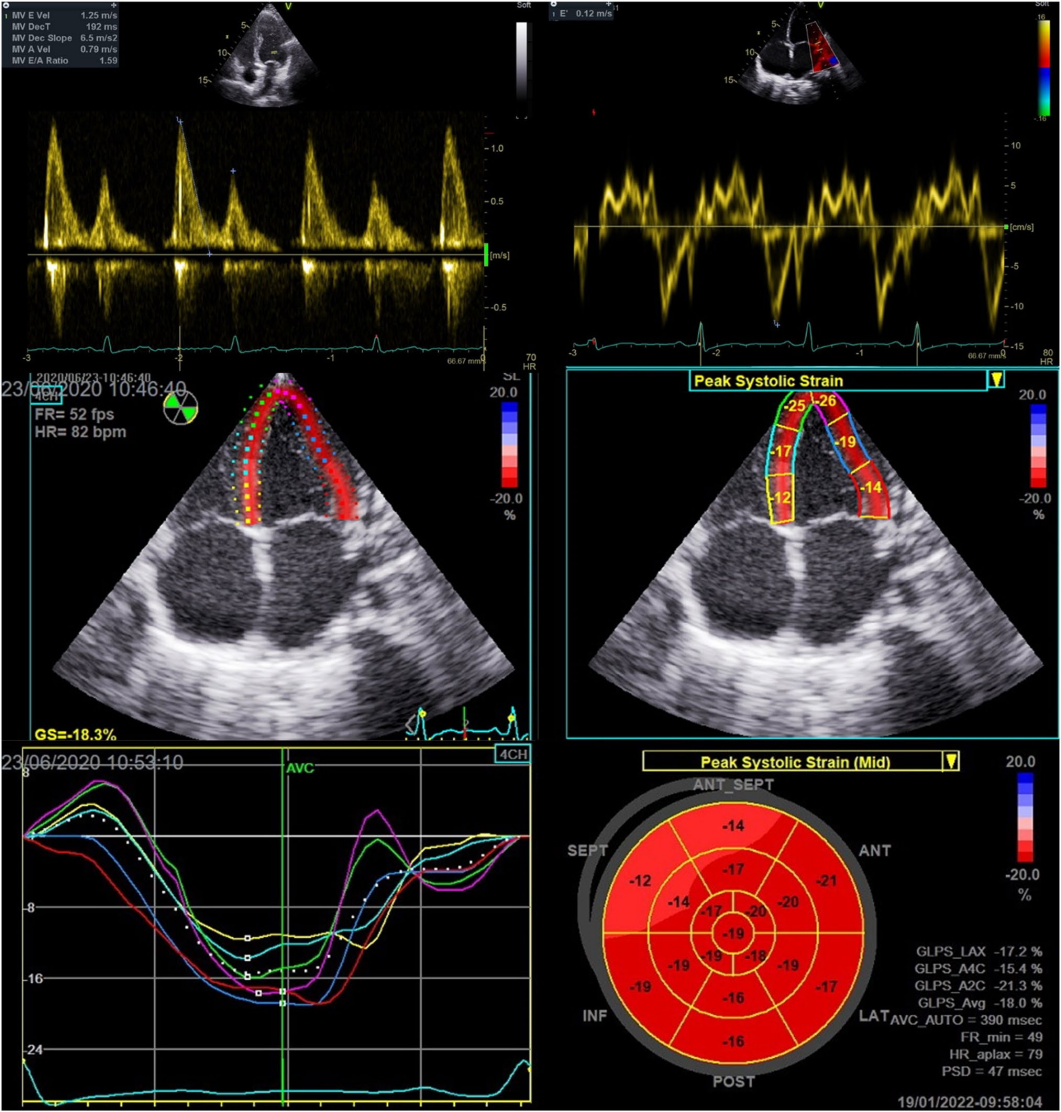
Doppler and strain echocardiographic images. *Top left*, pulsed-wave doppler recording at mitral inflow showing E, A velocities and E-wave DT. *Top right*, tissue doppler imaging at lateral mitral annulus showing e’. *Mid left*, region of interest tracing in the apical 4-chamber view during offline analysis using semi-automatic speckle-tracking software. *Mid right*, segments of left ventricle as seen in the apical 4-chamber view. *Bottom left*, peak systolic strain waveform obtained from apical 4-chamber view shown in mid panel. *Bottom right*, Bull's eye plot of longitudinal strain 3 apical views showing strain values of each segment

In the apical four chamber view, doppler echocardiography was used to assess peak early diastolic transmitral velocity (E), peak late diastolic transmitral velocity (A), E/A ratio, and mitral E-wave deceleration time (DT). Tissue doppler velocity analysis was done by placing a 2 mm doppler sample in septal and lateral mitral annulus to measure septal and lateral peak early diastolic velocity (e’). E/e’ was calculated as E divided by the mean of septal e’ and lateral e’ (Fig. 1) [12]. Right ventricular systolic pressure was estimated using the tricuspid regurgitation jet velocity.

#### Speckle tracking echocardiography

Strain and strain rate analysis was done offline using GE EchoPac software v201 (EchoPAC Advanced Analysis Technologies; GE Healthcare, US). All measurements were performed as recommended by the 2010 EACVI/ASE/Industry Task Force consensus document [13]. Two-dimensional speckle-tracking was performed using the three standard apical views by first manually tracing the endocardium at the onset of systole after which the acoustic tracking software tracked the myocardial speckle pattern frame-by-frame throughout the cardiac cycle. The region of interest was adjusted to cover the thickness of the myocardium and adequate tracking was verified and corrected if necessary. The aortic valve closure was identified on continuous wave doppler recording through the aortic valve. The LV was subsequently divided by the software into 18 segments covering the entire myocardium. Longitudinal strain was calculated for each of these segments and global longitudinal strain (GLS) was calculated as the average value of all 18 segments (Fig. 1). Similarly, global systolic strain rate (SR_S_) and global early diastolic SR (SR_E_) and late diastolic strain rate (SR_A_) were calculated from the average of all 18 segments. The E/SR_E_ ratio was calculated as the E velocity (m/s) divided by the global SR_E_ value.

At the end of the study, data on patient outcomes such as major adverse cardiovascular events (MACE), all-cause mortality and hospitalization with heart failure, were collected from hospital electronic medical records.

### Outcome definitions

*3-point MACE (3-P MACE)*: Composite of cardiovascular mortality, non-fatal myocardial infarction and non-fatal stroke.

*4-point MACE (4-P MACE)*: Composite of cardiovascular mortality, non-fatal myocardial infarction, non-fatal stroke and hospitalization for heart failure. Given the lack of accepted definition for heart failure in CKD population, we defined “hospitalization for heart failure” as symptoms of heart failure along with elevated N-terminal pro-B-type natriuretic peptide (NT-proBNP) levels (> 1200 pg/dL) requiring hospitalization and intravenous diuretic therapy and/or ultrafiltration for the purpose of this study [14].

### Statistical analysis

Statistical analysis was performed using SPSS version 22.0. Continuous data were tested for normality using the Kolmogorov–Smirnov test and those normally distributed were presented as mean and standard deviation (SD), while those not following normal distribution were expressed as median and interquartile range (IQR). Categorical data were presented as frequency (n) and percentages (%). Comparison of proportions was performed using the chi-squared test and normally distributed continuous data were compared using the two-sample t-test. Mann–Whitney U test was used to compare non-normal continuous data. All results were considered significant at a p-value of < 0.05.

## Results

### Patient characteristics

A total of 117 patients with CKD stages 3–5 were enrolled during the study period after obtaining written consent. The mean age of the study population was 57.3 ± 10.5 years, and 96 patients (82.1%) were male. The median serum creatinine was 3.5 (IQR, 2.2–6.0) mg/dL, with a median eGFR of 17.1 mL/min/1.73m^2^ (IQR, 9.0–29.9).

Fifty-six (47.9%) patients had biopsy-proven or presumed DKD, while 61 (52.1%) had CKD of other etiologies. The baseline characteristics of the study groups are shown in Table 1. There were significantly more hypertensives in the DKD group (98.2% vs. 85.2%; *p* = 0.017), however there was no difference in the baseline systolic and diastolic blood pressures. Higher number of patients in the DKD group were on statins (51.8% vs. 29.5%; *p* = 0.016) and anti-platelet drugs (44.6% vs. 21.3%; *p* = 0.010), compared to the NDKD group. There were no other significant differences between the two groups.

**Table 1:** Baseline characteristics of DKD and NDKD groups

Parameter	DKD (n = 56)	NDKD (n = 61)	*P* value
Demographics			
Age, years	59.1 ± 9.6	55.7 ± 11.1	0.078
Men	44 (78.6%)	52 (85.2%)	0.347
BMI, kg/m^2^	24.3 ± 3.6	23.5 ± 3.5	0.218
BSA, m^2^	1.7 ± 0.1	1.65 ± 0.1	0.158
Clinical Characteristics			
Etiology of CKD			
CGN	–	18 (29.5%)	
CIN	–	14 (23.0%)	
Others	–	7 (11.5%)	
Unknown	–	22 (36.1%)	
CKD stage			0.539
3	13 (23.2%)	16 (26.2%)	
4	20 (35.7%)	16 (26.2%)	
5	23 (41.1%)	29 (47.5%)	
Hypertension	55 (98.2%)	52 (85.2%)	**0.017**
PVD	1 (1.8%)	0 (0.0%)	0.479
CVA	1 (1.8%)	0 (0.0%)	0.479
Heart rate, min^−1^	85.7 ± 9.0	84.7 ± 9.3	0.572
Systolic BP, mmHg	138.0 ± 18.5	133.3 ± 20.1	0.195
Diastolic BP, mmHg	85.7 ± 7.5	84.0 ± 9.7	0.277
Medications			
ACEi/ARBs	5 (8.9%)	3 (4.9%)	0.477
Statins	29 (51.8%)	18 (29.5%)	0.016
Antiplatelets	25 (44.6%)	13 (21.3%)	0.010
Diuretics	30 (53.6%)	26 (42.6%)	0.269
Lab parameters			
eGFR, mL/min/1.73m^2^	20.5 ± 12.8	20.7 ± 15.0	0.924
Serum Creatinine, mg/dL	4.1 ± 2.4	5.1 ± 4.0	0.095
HbA1c, %	8.1 ± 2.3	–	**–**
UPCR, g/g	0.75 (0.46, 2.71)	0.78 (0.24, 2.41)	0.606
Serum calcium, mg/dL	8.0 ± 1.2	8.2 ± 1.2	0.414
Serum phosphorus, md/dL	4.4 ± 1.6	4.3 ± 1.8	0.868
PTH, pg/mL	251 ± 272	245 ± 153	0.951
TSH, mIU/mL	4.1 ± 5.6	2.9 ± 1.9	0.336

*ACEi/ARBs* angiotensin-converting enzyme inhibitors/angiotensin receptor blockers, *BMI* body mass index, *BP* blood pressure, *BSA* body surface area, *CGN* chronic glomerulonephritis, *CIN* chronic interstitial nephritis, *CKD* chronic kidney disease, *CVA* cerebrovascular accident, *eGFR* estimated glomerular filtration rate (as per CKD-EPI formula), *HbA1c* glycosylated hemoglobin, *PTH* parathyroid hormone, *PVD* peripheral vascular disease, *TSH* thyroid stimulating hormone, *UPCR* urine protein-creatinine ratio

### Echocardiographic findings

The main findings on traditional echocardiography are shown in Table 2. LV dimensions, including LV end-systolic and end-diastolic diameters, LV mass, LV mass index (LVMI), inter-ventricular septum (IVS) thickness and posterior wall thickness were similar in both groups. LV ejection fraction (LVEF), peak early diastolic transmitral velocity (E), peak late diastolic transmitral velocity (A) and E/A ratios were also similar in both groups. There was a significant difference in early diastolic mitral annular velocity at interventricular septum (septal e’) (7.1 ± 2.5 vs. 8.2 ± 2.8; *p* = 0.031) and lateral wall (lateral e’) (9.2 ± 2.9 vs. 10.4 ± 3.8; *p* = 0.045) measured by tissue doppler imaging in the DKD and NDKD groups. The E/e’ ratio was significantly higher in DKD (11.9 ± 4.4 vs. 9.8 ± 3.5; *p* = 0.004) and so was the mitral E wave deceleration time (in milliseconds) (209.2 ± 41.5 vs. 189.1 ± 48.0; *p* = 0.017), compared to those with NDKD. Overall, abnormal E/e’ (> 12) was seen in 40 (34.2%) patients. Higher proportion of patients with DKD had abnormal E/e’ compared to those with NDKD (44.6% vs 24.6%; *p* = 0.022).

**Table 2:** Conventional echocardiographic parameters of DKD and NDKD groups

Parameter	DKD (n = 56)	NDKD (n = 61)	*P* value
Dimensions			
IVS thickness, mm	13.2 ± 1.9	12.6 ± 2.3	0.104
PW thickness, mm	12.2 ± 1.8	11.6 ± 1.9	0.079
LVEDD, mm	48.1 ± 5.6	47.2 ± 4.4	0.353
LVESD, mm	30.5 ± 5.4	29.8 ± 3.8	0.385
LVEDV, mL	88.6 ± 20.3	85.8 ± 18.1	0.426
LVESV, mL	33.8 ± 13.1	30.3 ± 8.3	0.089
LVMI, g/m^2^	145.2 ± 42.9	132.7 ± 37.1	0.099
RWT	0.511 ± 0.085	0.495 ± 0.097	0.365
Systolic			
FS, %	36.8 ± 5.3	37.0 ± 8.2	0.901
LVEF, %	65.7 ± 5.0	66.1 ± 4.2	0.608
TAPSE, mm	2.1 ± 0.3	2.1 ± 0.2	0.274
RVSP, mmHg	26.4 ± 10.5	29.1 ± 11.1	0.171
Diastolic			
E (mitral), cm/s	90.3 ± 24.9	84.9 ± 24.3	0.241
A (mitral), cm/s	89.1 ± 23.3	81.7 ± 26.0	0.109
E/A	1.12 ± 0.6	1.29 ± 0.5	0.426
e’ (septal), cm/s	7.1 ± 2.5	8.2 ± 2.8	**0.031**
e’ (lateral), cm/s	9.2 ± 2.9	10.4 ± 3.8	**0.045**
DT, ms	209.2 ± 41.5	189.1 ± 48.0	**0.017**
E/e’	11.9 ± 4.4	9.8 ± 3.5	**0.004**

All significant p-values, ie. p-value < 0.05 have been highlighted in bold

A, peak late Diastolic transmitral velocity; *DT* mitral E wave deceleration time, *E* peak early diastolic transmitral velocity, e’, early diastolic mitral annular velocity, *FS* fractional shortening, *IVS* interventricular septum, *LVEDD* left ventricular end-diastolic diameter, *LVEDV* left ventricular end-diastolic volume, *LVEF* left ventricular ejection fraction, *LVESD* left ventricular end-systolic diameter, *LVESV* left ventricular end-systolic volume, *LVMI* left ventricular mass index, *PW* posterior wall of left ventricle, *RVSP* right ventricular systolic pressure, *RWT* relative wall thickness, *TAPSE* tricuspid annular plane peak systolic excursion

### Strain echocardiographic findings

There was no significant difference in the average GLS of both groups (Table 3). Low GLS (> −16%) was seen in 15 (26.8%) patients with DKD and 20 (32.8%) with NDKD (*p* = 0.479). E/e’ ratio was higher in patients with low GLS compared to those with normal values (12.5 ± 5.1 vs. 10.0 ± 3.3; *p* = 0.002).

**Table 3:** Strain echocardiography parameters of DKD and NDKD groups

Parameter	DKD (n = 56)	NDKD (n = 61)	*P* value
Systolic			
GLS, %	−18.1 ± (−4.0)	−18.1 ± (−4.0)	0.997
SR_S_, s^−1^	−1.2 ± (−0.3)	−1.2 ± (−0.3)	0.600
Diastolic			
SR_E_, s^−1^	1.3 ± 0.4	1.4 ± 0.4	0.663
SR_A_, s^−1^	1.1 ± 0.4	1.2 ± 0.5	0.742
E/SR_E_*10	73.9 ± 32.4	66.8 ± 24.9	0.183

E/SR_E_, ratio of peak early diastolic transmitral velocity to early diastolic strain rate; *GLS* global longitudinal strain of left ventricle, *SR*_*A*_ late diastolic strain rate, *SR*_*E*_ early diastolic strain rate, *SR*_*S*_ peak systolic strain rate

The LV peak systolic strain rate (SR_S_), LV early diastolic strain rate (SR_E_) and LV late diastolic strain rate (SR_A_) were similar across both groups. There was no statistically significant difference in the E/SR_E_ ratios of both groups, although it was numerically higher in the DKD group (73.9 ± 32.4 vs. 66.8 ± 24.9, *p* = 0.183).

### Clinical outcomes

At the end of a median follow-up of 239 (IQR, 76.5–566) days, those with DKD had higher 3-P MACE (11.5% vs. 4.9%; *p* = 0.047) and 4-P MACE (28.6% vs. 11.5%; *p* = 0.020), compared to those with NDKD (Table 4.). Other clinical outcomes such as all-cause mortality and cardiovascular mortality were similar in both groups.

**Table 4:** Clinical outcomes of DKD and NDKD groups

Parameter	DKD (n = 56)	NDKD (n = 61)	*P* value
3-point MACE	9 (11.5%)	3 (4.9%)	**0.047**
Non-fatal MI	5 (8.9%)	2 (3.3%)	0.257
Non-fatal stroke	0 (0%)	1 (1.6%)	1.00
CV death	4 (7.1%)	0 (0%)	**0.049**
4-point MACE	16 (28.6%)	7 (11.5%)	**0.020**
HHF	7 (12.5%)	4 (6.6%)	0.348
All-cause death	5 (8.9%)	4 (6.6%)	0.735
CV death	4 (7.1%)	0 (0%)	**0.049**

All significant p-values, ie. p-value < 0.05 have been highlighted in bold

*CV* cardiovascular, *HHF* hospitalization for heart failure, *MACE* major adverse cardiovascular events

## Discussion

We studied echocardiographic parameters of patients with CKD stages 3–5 and normal ejection fraction and found that the ratio of peak early diastolic transmitral velocity to early diastolic mitral annular velocity (E/e’) was abnormal in nearly a third of patients. Further, E/e’ was higher in patients with DKD, compared to those with NDKD (11.9 ± 4.4 vs. 9.8 ± 3.5; *p* = 0.004). E/e’ ratio is an estimate of LV end diastolic pressure and is widely considered to be a non-invasive marker of LV diastolic dysfunction [15]. We also found that both septal e’ (7.1 ± 0.25 vs. 8.2 ± 2.8; *p* = 0.031) and lateral e’ (9.2 ± 2.9 vs. 10.4 ± 3.8; *p* = 0.045) were significantly lower in the DKD group, in addition to higher mitral E wave deceleration times (209.2 ± 41.5 vs. 189.1 ± 48.0; *p* = 0.017). While the lower septal and lateral e’ values indicate impairment in LV relaxation and early diastolic recoil, longer DT indicates LV stiffness-these are the key underlying mechanisms for diastolic dysfunction [16]. Despite the fact that both groups had similar LVMI and systolic function, the observed differences in the indices of LV diastolic function suggest that there are additional mechanisms at play in patients with DKD compared to those with other etiologies for CKD. In a study similar to ours, Miyazato et al. reported that echocardiographic parameters of LV diastolic function were more impaired in those with diabetic nephropathy, compared to those with chronic glomerulonephritis [17]. There too the authors observed no difference in LV structure and systolic function between the two groups. Another study by Han et al*.* too found higher E/e’ ratios in patients with DKD compared to those with NDKD [18]. It has been described that the diabetic state is associated with increased oxidative stress, elevated levels of pro-fibrotic and pro-inflammatory cytokines and accumulation of advanced glycation end products [19]. These abnormalities can lead to myocyte apoptosis, fibrosis, and LVH resulting in a condition referred to as diabetic cardiomyopathy-an entity that is still poorly understood [20–22]. This condition manifests as diastolic dysfunction in the presence of preserved EF in the initial stages, with systolic dysfunction developing over time. Some of these factors like LVH and fibrosis are also operative in non-diabetic CKD [3, 23]. However, in addition, diabetic state is also associated with abnormal myocyte relaxation due to abnormal calcium handling in cardiomyocytes [24]. Prognostically, the presence of diastolic dysfunction assumes importance since evidence suggests that it is associated with higher all-cause and cardiovascular mortality [25]. Even in our study, patients with DKD had poorer cardiovascular outcomes in a relatively short follow-up period. These findings make a strong argument for the routine assessment of diastolic function in patients with DKD.

The role of LV deformation imaging (using speckle tracking echocardiography) for early detection of diastolic dysfunction remains unclear at present. Like e’, SR_E_ is a marker of LV relaxation in early diastole. However, technical difficulties and lack of reference standards make the interpretation of diastolic parameters challenging [16]. It has been suggested that the ratio of peak early diastolic transmitral velocity to early diastolic strain rate (E/SR_E_) may be better than E/e’ ratio for detection of LV diastolic dysfunction in certain patient populations [26]. E/SR_E_ has also been described to be a superior to E/e’ for prediction of MACE in the hemodialysis population [27, 28]. In our study, however, we found no statistical difference between E/SR_E_ of the DKD and NDKD groups, although E/e’ was significantly different. Of note, E/ SR_E_ was numerically higher in the DKD group compared to NDKD (73.9 ± 32.4 vs. 66.8 ± 24.9; *p* = 0.183) and so the lack of statistical difference could be explained by the relatively small sample size. Moreover, the role of E/SR_E_ in pre-dialysis CKD has not been studied previously and is still uncertain.

A low GLS (> − 16%) suggestive of LV systolic dysfunction was seen in approximately 30% of our study population even though the study included only those with a normal LVEF. This confirms the findings of previous studies that strain imaging-derived GLS precedes changes in EF observed by conventional echocardiography [29–31]. GLS has been shown to be a predictor of all-cause and cardiovascular mortality in several studies [7, 32, 33]. However, there was no difference in GLS between the DKD and NDKD groups in our study. This indicates that, although deformation imaging is able to identify the so-called “subclinical LV systolic dysfunction” in almost a third of these patients, DKD does not seem to be associated with a worse systolic function compared to those with NDKD in our study. Further, we found that patients with a low GLS (> − 16%) had higher E/e’ compared to those with normal GLS (12.6 ± 5.1 vs. 10.0 ± 3.3; *p* = 0.002). Although conventional wisdom is that GLS is a marker of subclinical LV systolic dysfunction, recent studies have suggested that it may provide additional information. A study by DeVore et al., showed that GLS may be a marker of myocardial fibrosis and hence diastolic dysfunction by showing an association between abnormal GLS and higher levels of biomarkers of collagen synthesis and NT-proBNP in blood [34]. The finding in our study that those with abnormal GLS had higher E/e’ (indicating diastolic dysfunction) suggests myocardial fibrosis as a possible explanation for impaired diastolic function. However, it remains unclear as to why DKD group had higher E/e’ compared to NDKD group even though both groups had similar proportion of patients with impaired GLS. It is possible that apart from myocardial fibrosis, there are additional factors such as abnormal myocardial relaxation (as described above) that account for higher E/e’ in the DKD group.

There were no differences in peak systolic strain rates (SR_S_) between DKD and NDKD. As with diastolic strain rates, the lack of standardization in measurement techniques and cut-offs pose a challenge in the analysis of these newer parameters.

At the end of a median follow-up of 239 days, higher rates of 3-P MACE (11.5% vs. 4.9%; *p* = 0.047) and 4-P MACE (28.6% vs. 11.5%; *p* = 0.020) were observed in the DKD group, indicating poorer outcomes even in the short-term. Clearly, these two groups are clinically different in terms of cardiovascular outcomes even in the relatively short follow up period of our study. But the only difference with respect to echocardiography was in diastolic function. Both groups had similar systolic function even when sensitive parameter like GLS was used. Therefore, it is plausible that diastolic dysfunction could be one of the factors associated with future cardiovascular events. However, the number of events in our study is small and it is difficult to draw conclusions on the association of diastolic dysfunction (and other echocardiographic parameters) and clinical outcomes.

This study is unique on two counts. Firstly, we used speckle-tracking echocardiography, a more sensitive modality, to identify subtle differences in cardiac function in the DKD and NDKD groups. Secondly, we specifically included CKD patients with normal EF (in contrast to previous studies) because indices of diastolic function can be affected once left ventricular EF is reduced and may not necessarily indicate diastolic dysfunction per se. Moreover, patients with reduced EF represent those with a more advanced stage of cardiac dysfunction. Here, we attempted to identify early echocardiographic changes prior to the onset of frank LV systolic dysfunction by including only those with normal EF. The limitations of this study are the short duration of follow-up and the small sample size. Approximately one-fourth of patients in the NDKD group had diabetes mellitus. Although none of them had any other diabetes-related target-organ damage such as diabetic retinopathy, neuropathy or atherosclerotic cardiovascular disease at the time of inclusion in the study, the influence of diabetes on the myocardium in this group cannot be ruled out. Those with recent-onset diabetes may not have undergone myocardial remodeling and therefore, the duration of the disease could also have affected the findings. Moreover, a higher proportion of patients in the DKD group had hypertension, compared to the non-DKD group and this may have impacted echocardiographic findings and patient outcomes, even though mean systolic and diastolic blood pressures were similar in both groups at the time of entry into the study. Data on novel echocardiographic parameters such as LA strain (which is increasingly being recognized as a useful tool to assess diastolic dysfunction) and layer-specific strain is also lacking [35, 36]. Finally, this being a single-center study conducted in the Indian population, the generalizability of our findings may be limited.

### Conclusions

Lower e’, longer DT and higher E/e’ was observed in DKD patients compared to those with NDKD suggesting diastolic dysfunction was more common in DKD at a stage when EF was normal. However, LV GLS, which was abnormal in 30% of the overall cohort, was comparable among DKD and NDKD groups. Cardiovascular outcomes were also worse in the DKD group. Further studies are needed to determine if cardiomyopathy in DKD represents a distinct phenotype that carries a prognostic implication different from that of cardiomyopathy in other etiologies of CKD. The impact of these echocardiographic differences on therapeutic approaches also needs to be explored.
